# Multifocal Pseudomyogenic Hemangioendothelioma Involving the Scalp and Nose, Misdiagnosed as A Sarcoma: A Rare Case Report

**DOI:** 10.5146/tjpath.2021.01539

**Published:** 2022-01-21

**Authors:** Neha Mittal, Bharat Rekhi, Priyamvada Singhal, Munita Bal, Swapnil Rane, Asawari Patil, Shivakumar Thiagarajan

**Affiliations:** Department of Surgical Pathology, Tata Memorial Hospital, Mumbai, India; Subharti Medical College, Uttar Pradesh, India; Department of Head and Neck Surgical Oncology, Tata Memorial Hospital, Mumbai, India

**Keywords:** Hemangioendothelioma, Soft tissue neoplasms, Epithelioid cells, Scalp, Sarcoma

## Abstract

This case report aims to present clinicopathological features of an extremely rare case of multifocal pseudomyogenic hemangioendothelioma (PMHE) in the scalp.

A 21-year-old male developed multiple, focally ulcerated, nodules over the root of his nose and scalp. One of the skin lesions was sampled at another dermatology clinic, where this was diagnosed as a sarcoma. A review of biopsy sections showed well-circumscribed dermal lesions, comprising plump spindle and epithelioid cells, mimicking rhabdomyoblasts. Immunohistochemically, tumor cells were positive for AE1/AE3, CD31, FLI-1 and ERG. INI-1 was retained. A diagnosis of PMHE was offered. Subsequently, the patient underwent wide excision and has been asymptomatic for 8 months, post-surgery.

PMHE is rarely reported in the head and neck region, where it can constitute a diagnostic pitfall. Awareness of this tumor and appropriate immunohistochemical stains are necessary for its timely diagnosis, in order to avoid radical treatments. A review of similar, previously documented cases is presented.

## INTRODUCTION

Pseudomyogenic haemangioendothelioma (PMHE), previously termed as epithelioid sarcoma-like hemangioendothelioma, is currently defined as an intermediate malignant, rarely metastasizing neoplasm, displaying vascular/endothelial differentiation. It mostly occurs in the soft tissues of lower extremities of young adult males. Histopathologically, PMHE simulates high-grade sarcomas, such as spindle cell rhabdomyosarcoma and epithelioid sarcoma (1–3). It has rarely been described in the bones (4,5). The head and neck region constitutes one of the rarest sites (2,3,5–9).

## CASE REPORT

A 21-year-old male presented to a dermatologist with multiple nodular lesions involving the skin of his scalp, forehead, and root of the nose of 4 months duration. He underwent a biopsy from the lesion over his nose which, at two different laboratories, was reported as high-grade sarcoma and leiomyosarcoma, respectively.

**Figure 1:**
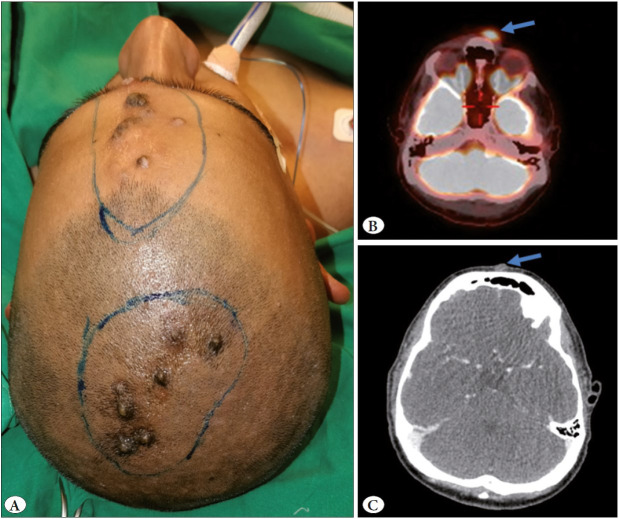
**A)** Clinical photograph showing multiple, grouped, fleshy, cutaneous lesions, including a few ulcerated, involving the scalp and the root of the nose. **B)** Positron emission (PET) scan showing FDG-avid multiple nodular cutaneous lesions involving the nasal root (arrow), and frontal and left parietal region. **C)** PET-CT scan displaying an isointense lesion involving the skin and subcutaneous tissues on the root of the nose (arrow), sparing the underlying bone.

Thereafter, he was referred to us. During clinical evaluation, he seemed to be in good general health. During local examination, there were multiple, painless, nodular, fleshy lesions involving the skin of his scalp, forehead, and nose (Figure 1A). A clinical diagnosis of cutaneous sarcoma was considered. He underwent radiological evaluation and the slides and paraffin blocks of his nasal lesion were reviewed.

Positron emission tomography (PET) scan showed multiple FDG-avid lesions involving the nasal root, and the frontal and left parietal region, the largest measuring 12 mm x 7.9 mm (SUVmax = 7.02) (Figure 1B, C). A wide local excision of the lesions, followed by a reconstruction with a free flap was undertaken.

During review, the tumor sections were tested for various immunohistochemical antibody markers on an automated Ventana Benchmark XT® platform.

### Gross Findings

The resection specimens of the root of the nose and posterior scalp lesions were well-oriented, measuring 9 cm x 4.5 cm x 0.5cm and 8.5 cm x 5.5 cm x 0. 6 cm, respectively. Cut surfaces of both the specimens showed multiple grey-white, focally ulcerating, fleshy lesions.

### Histopathological Findings

Microscopically, the biopsy revealed an infiltrating tumor, composed of spindle-shaped cells, involving the dermis and subcutaneous tissue.

**Figure 2:**
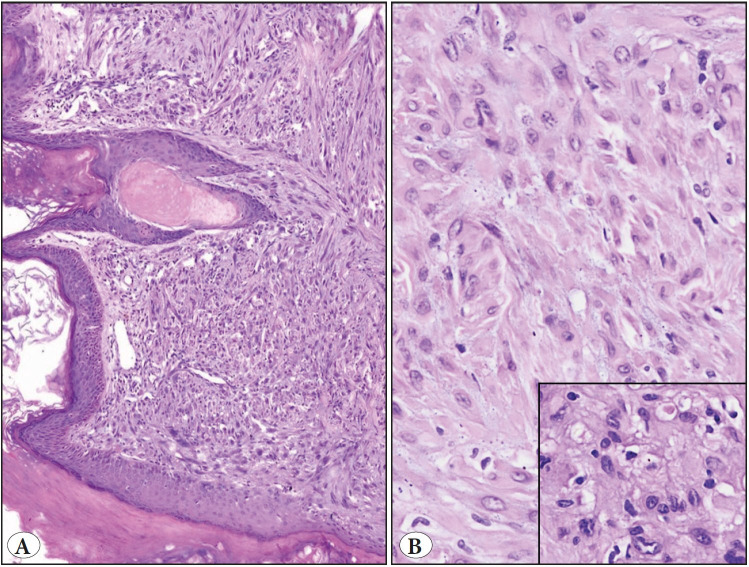
Microscopic features. **A)** A cellular spindle cell tumor, involving the dermis with cells arranged in interlacing fascicles (H&E, x100). **B)** Tumor composed of plump spindle-shaped cells with vesicular nuclei and abundant eosinophilic cytoplasm, reminiscent of a myoid appearance with few interspersed neutrophils (H&E, x200). Inset: Higher magnification showing cells with an abundant eosinophilic cytoplasm and vesicular nuclear chromatin (H&E, x400).

Microscopic examination of the resection specimens revealed infiltrative, focally well-demarcated lesions, appearing as granulomas in lower magnification. The tumor cells were arranged in interlacing fascicles (Figure 2A). On higher magnification, tumor cells were seen insinuating in between the adnexal structures and dermal nerves. Individual tumor cells were plump, spindle-shaped, containing moderate to abundant eosinophilic cytoplasm; central to eccentrically placed vesicular nuclei and discernible nucleoli, reminiscent of a “myoid” appearance. There were few bi- and multi-nucleate forms. Focal areas displayed “rhabdoid-like” cells, containing eccentrically placed crescentic nuclei and paranuclear eosinophilic inclusions. Additionally, there were pseudo-microcysts comprising cells with intracytoplasmic vacuoles. There were no significant mitotic figures and/or necrosis (Figure 2A,B).

**Figure 3:**
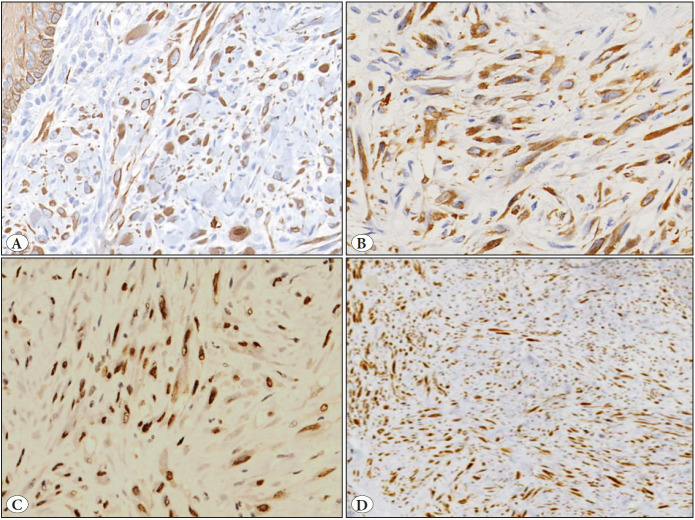
Immunohistochemical results. **A)** Tumor cells displaying diffusely positivity for AE1/AE3. (AE1/AE3 antibody, x100). **B)** Tumor cells showing CD31 positivity (CD31 antibody, x400). **C)** Diffuse FLI-1 positivity (FLI-1 antibody, x400). **D)** Tumor cells showing retained INI1 immunostaining (INI1 antibody, x200).

**Figure 4:**
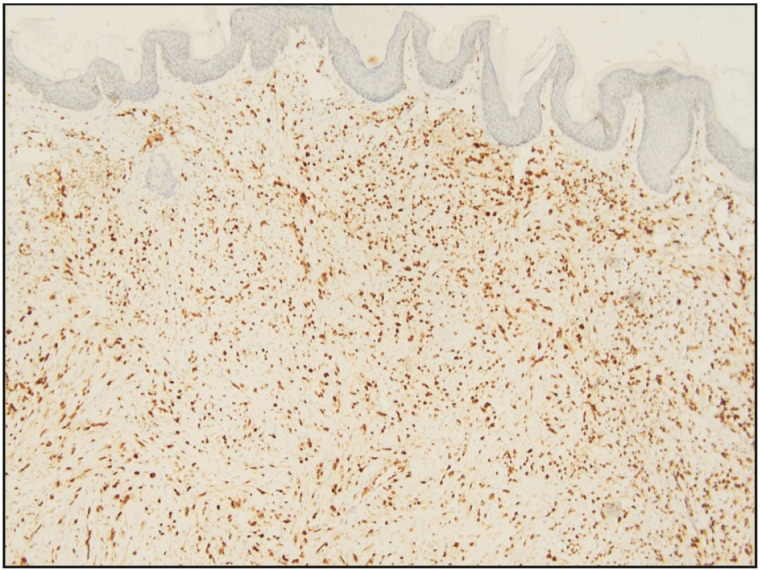
Tumor cells displaying diffuse ERG positivity (ERG antibody, x 200).

Immunohistochemically, tumor cells showed positivity for AE1/AE3, FLI-1, CD31 and ERG, and negativity for smooth muscle actin (SMA), desmin, CD 34, P40, CD1a, S100 P and CD163. INI1/SMARCB1 was diffusely retained (Figure 3A-D) (Figure 4). Ki67/Mib1 highlighted 8-10% tumor cell nuclei in the highest proliferating areas. All the resection margins and base of both the resection specimens were free of tumor.

## DISCUSSION

Multifocal pseudomyogenic hemangioendothelioma (PMHE) is characterized by young age, male predisposition, extremity location, multifocality and dermal location. Microscopically, it displays infiltrative borders and tumor cells resembling rhabdomyoblasts (3). Till date, only 12 cases of PMHE have been reported in the head and neck region, including 2 cases in the scalp, as noted in the present case (2,3,5–9). Other reported uncommon sites include mucosal involvement in a single case and lymph node metastasis in 2 cases, of the head and neck region (Table 1) (7–9).

**Table 1:** Summary of clinicopathological parameters of cases of PMHE, reported in the head and neck region.

**Authors, Year**	**Age/Sex**	**Location**	**Multifocal**	**Size (cm)**	**Tissue planes**	**Initial diagnosis (Clinical/histological)**	**Treatment**	**Recurrence**	**Metastasis**	**Follow up in months**
Billings et al, 2003 (2)	45/F	Scalp	NK	<2	NK	Epithelioid sarcoma	WLE	Yes, 36mo	No	NED after 2nd WLE
Hornick and Fletcher, 2011 (3)	47/M	Nose	Yes	NK	D	NK	NK	NK	NK	NK
Hornick and Fletcher, 2011 (3)	34/M	Forehead	No	0.6	SC	NK	NK	NK	NK	NK
Cai et al, 2011 (5)	-	Neck	NK	NK	NK		NK	NK	NK	NK
Requena et al, 2013 (6)	27/M	Perioral	Yes	NK	D	Molluscum contagiosum	WLE	No	No	NED, 12
Rawal et al, 2017 (7)	21/F	Oral cavity	No	1.4	Mucosa	Pyogenic granuloma	WLE	No	No	NED, 24
Ijzendoorn et al, 2018 (8)	17/M	Skin of head, neck and nodes	Yes	NK	SC	NK	Chemotherapy, f/b Targeted therapy by Telatinib	3 months on chemotherapy, NED after Telatinib	Yes	NED (3mo), 108
Hung et al, 2017 (9)	NK	Skull(n=2), Face(n=2) and intraparotid node	NK	NK	NK	NK	NK	NK	Yes, Intraparotid node	NK
Present case, 2020	21/M	Root of nose and scalp	Yes	1.8	SC	Leiomyosarcoma and High grade sarcoma, NOS	WLE	No	No	NED, 24

**M:** Male, **F:** Female, **NK:** Not known, **D:** Dermis, **WLE:** Wide local excision, **SC:** Subcutaneous, **NK:** Not Known, **NED:** No evidence of disease

Lack of overt vascular differentiation and a close resemblance, both clinically, as well as histologically, to other relatively commoner sarcomas, along with unawareness of this entity might mislead an unwary pathologist, as noted in the present case, which was misdiagnosed at two different laboratories.

In view of its overlapping features with other sarcomas, certain immunohistochemical antibody markers, including cytokeratins and vascular markers, along with INI1/SMARCB1 are essential for its diagnosis (3). A range of differential diagnoses have been considered in earlier reported cases, including an epithelioid sarcoma, rhabdomyosarcoma, epithelioid hemangioendothelioma, and sarcoma, not otherwise specified (NOS). The closest differential diagnosis in the present case was an epithelioid sarcoma, in view of a relatively superficial location, younger age and immunohistochemical expression of cytokeratin (6,8). However, a predominantly spindle cell morphology, along with immunopositivity for CD31 and retained expression of INI1, ruled out this possibility (3,7,8). Positivity for epithelial antibody markers and negative staining for skeletal muscle-specific markers, namely desmin, myogenin and MyoD1, ruled out a spindle cell rhabdomyosarcoma. It is noteworthy that most of the differential diagnoses of a PMHE are high-grade and relatively aggressive tumors.

Apart from its distinct morphological features and immunohistochemical profile, PMHE is characterized by a distinct molecular signature, in the form of a genetic fusion between *FOSB* (19q) with a strong promoter, *SERPINE* (7q22), *ACTB*(7p22), and recently *WWTR1*(3q25), all of which lead to an upregulation of *FOSB* expression (9–11). This led to development of FOSB immunostain for the diagnosis of PMHE (9). At the same time, FOSB is also expressed in epithelioid haemangiomas and in osteoblastomas. In view of unavailability, we could not test our case with FOSB. Nonetheless, unequivocal histopathological features supplemented with necessary immunohistochemical markers were supportive of a diagnosis of PMHE (Table 2).

**Table 2:** Results of immunohistochemical (IHC) markers tested in various cases of PMHE of the head and neck region, as reported in the literature.

**IHC results**	**AE1/AE3**	**CD31**	**FLI1**	**ERG**	**SMA**	**Desmin**	**CD34**	**INI1**	**S100 protein**	**Other IHC markers**
Billings et al (2)	+	+	+	NK	NK	NK	-	R	NK	Vim +
Hornick and Fletcher (3)	+	NK	+	ND	NK	-	-	R	-	-
Hornick and Fletcher (3)	+	NK	+	ND	NK	-	-	R	-	-
Cai et al (5)	+	+	+	ND	ND	ND	NK	NK	NK	-
Requena et al (6)	+	+	+	+	Focal, weak+	-	-	R	-	Myogenin-, MyoD1 -
Rawal et al (7)	+	+	ND	ND	-	-	-	ND	-	HMB-45-
Ijzendoorn et al (8)	NK	NK	NK	NK	NK	NK	NK	NK	NK	FOSB
Hung et al (9)	NK	NK	NK	NK	NK	NK	NK	NK	NK	FOSB +
Present case	+	+	+	+	Focal, weak+	-	-	R	-	P40 -

+: Positive, -: Negative, NK: Not known, ND: Not Done, R: Retained

Contrary to some of its worrisome histopathological features, PMHE has an indolent clinical course with potential for local recurrence and a minimal risk of distant metastasis. Disseminated disease and metastasis has been reported in 6% of the cases (9). Development of metastasis after 5 years of the initial diagnosis, as previously reported, implies the need for a prolonged follow-up in such cases (3). The treatment is mainly aimed at wide excision of the lesions, instead of radical resections and adjuvant therapies, which are considered for its mimics such as an epithelioid sarcoma, malignant rhabdoid tumor and a spindle cell rhabdomyosarcoma. Adjuvant local radiotherapy has been offered in unresectable cases of PMHE. The present case was treated with wide-excision. The patient is alive with no evidence of disease, 8 months post-surgery.

In conclusion, PMHE is an uncommon, locally aggressive tumor of abstruse vascular origin, and an uncertain malignant potential affecting dermal and or subcutaneous tissue of various anatomical sites in young males. The head and neck, especially nose and scalp constitute its rare sites. With only 12 such reported cases in world literature, the present case, including its clinical impact, seemed worth reporting. Awareness of this entity, careful assessment of morphological features and certain immunohistochemical markers are necessary for its correct and timely diagnosis, in order to obviate unnecessary radical treatments, the latter reserved for recurrences or unresectable tumors, which in itself are exceedingly rare in these tumors.

## Conflict of Interest

The authors declare no conflict of interest.
